# How differing methods of ascribing ethnicity and socio-economic status affect risk estimates for hospitalisation with infectious disease

**DOI:** 10.1017/S0950268818002935

**Published:** 2018-11-13

**Authors:** Mark R. Hobbs, Polly Atatoa Carr, Jacinta Fa'alili-Fidow, Avinesh Pillai, Susan M. B. Morton, Cameron C. Grant

**Affiliations:** 1 Growing Up in New Zealand, Centre for Longitudinal Research, University of Auckland, Private Bag 92019, Auckland, New Zealand; 2 Infectious Diseases Department, Auckland City Hospital, Auckland District Health Board, Auckland, New Zealand; 3 National Institute Demographic Economic, Faculty of Arts and Social Sciences, University of Waikato, Hamilton, New Zealand; 4 Department of Statistics, Faculty of Science, University of Auckland, Auckland, New Zealand; 5 Department of Paediatrics, Faculty of Medicine and Health Sciences, Child & Youth Health, University of Auckland, Auckland, New Zealand; 6 General Paediatrics, Starship Children's Hospital, Auckland District Health Board, Auckland, New Zealand

**Keywords:** Ethnicity/race, infectious disease epidemiology, paediatrics, socio-economic status, statistics

## Abstract

Significant ethnic and socio-economic disparities exist in infectious diseases (IDs) rates in New Zealand, so accurate measures of these characteristics are required. This study compared methods of ascribing ethnicity and socio-economic status. Children in the *Growing Up in New Zealand* longitudinal cohort were ascribed to self-prioritised, total response and single-combined ethnic groups. Socio-economic status was measured using household income, and both census-derived and survey-derived deprivation indices. Rates of ID hospitalisation were compared using linked administrative data. Self-prioritised ethnicity was simplest to use. Total response accounted for mixed ethnicity and allowed overlap between groups. Single-combined ethnicity required aggregation of small groups to maintain power but offered greater detail. Regardless of the method used, Māori and Pacific children, and children in the most socio-economically deprived households had a greater risk of ID hospitalisation. Risk differences between self-prioritised and total response methods were not significant for Māori and Pacific children but single-combined ethnicity revealed a diversity of risk within these groups. Household income was affected by non-random missing data. The census-derived deprivation index offered a high level of completeness with some risk of multicollinearity and concerns regarding the ecological fallacy. The survey-derived index required extra questions but was acceptable to participants and provided individualised data. Based on these results, the use of single-combined ethnicity and an individualised survey-derived index of deprivation are recommended where sample size and data structure allow it.

## Introduction

The epidemiology of infectious disease (ID) in New Zealand (NZ) is marked by significant ethnic and socio-economic disparities, with higher rates observed in Māori and Pacific peoples, and in areas of greater socio-economic deprivation.[1–3] Likewise, higher rates of ID are also seen in indigenous and marginalised ethnic minority groups in comparable developed countries such as Australia and the USA [4, 5]. Therefore, accurate measures of ethnic identity and socio-economic deprivation are of particular importance in epidemiological research.

Ethnicity is a complicated social construct that describes cultural identity or affiliation [6]. The related but distinct concept of race is not generally used in research in NZ. There are a number of methods of ascribing ethnicity in epidemiological research including self-prioritisation, total response and single-combined ethnicity, each method having its advantages and disadvantages [7]. Socio-economic deprivation can also be measured in various ways, including by directly questioning household income, and by using census-derived geographic measures [8] or survey-derived individual measures [9].

In this study, we used hospitalisation for an ID before the age of 5, an outcome known to be linked to ethnicity and socio-economic disadvantage [2], to compare different measures of ethnicity and socio-economic deprivation.

## Methods

### Study population

This study was conducted within the *Growing Up in New Zealand* (GUiNZ) longitudinal birth cohort. GUiNZ enrolled 6822 pregnant mothers from the Auckland, Counties-Manukau and Waikato District Health Board areas due to deliver in 2009–2010 [10, 11]. The cohort includes 6853 children born from these pregnancies, 11% of the national birth cohort. The cohort is generalisable to the national birth cohort with regards to ethnic and socio-economic diversity [12].

### Ethics

Ethical approval was obtained from the Ministry of Health Northern Y Regional Ethics Committee (NTY/08106/055). Written informed consent for interviews and data linkage was completed by each child's primary caregiver.

### Data collection and linkage

GUiNZ has conducted computer-assisted face-to-face interviews with the primary caregiver of each study child at multiple time points, including during the antenatal period, and at 9 months, 2 and 4½ years of child age. This study uses data from the interview conducted at 4½ years of child age. Linkage was established between GUiNZ datasets and the National Minimum Dataset (NMDS) using each child's unique National Health Index number. The NMDS is a national administrative health dataset that contains records of all public hospital admissions in NZ, including emergency department visits but not outpatient clinic attendances [13]. Public hospital care, including all acute inpatient paediatric care, is free for NZ permanent residents and citizens. The NMDS includes discharge diagnoses coded using the Australian Modification of the International Classification of Diseases and Health Related Problems (ICD-10-AM) [14].

### Primary outcome

The primary outcome was hospitalisation for an ID in the first 5 years of life, treated as a binary (ever/never) variable. Hospitalisations for an ID were identified using the first diagnostic code listed in the NMDS, as the first listed code represented the primary health problem managed during that hospitalisation. This was done to avoid counting nosocomial infections and minor infections, where infection was not the main reason for hospitalisation. Acute and chronic viral, bacterial, fungal and parasitic infections were included, as were common infective syndromes without a specific microbiological diagnosis, such as fever, upper respiratory tract infection and gastroenteritis. Infective exacerbations of chronic non-infective conditions such as asthma were included but hospitalisations for non-infective sequelae of past infections were excluded. Birth hospitalisations and hospitalisations for non-medical reasons (e.g. maternal hospitalisation) were also excluded.

### Ethnicity variables

Primary caregivers were asked the following questions regarding the ethnicity of the study child:

• **Total response ethnicity question:** Which ethnic group or groups does (child name) belong to?
• **Prioritised ethnicity question:** Which is the MAIN ethnic group that (child name) identifies with?

Multiple responses were allowed for the total response ethnicity question. A single response was requested for the prioritised ethnicity question, but if this was not possible, up to two responses were allowed. For both questions, caregivers were offered a list of 32 of the most common ethnicities in NZ, and could also choose to specify up to two unlisted ethnicities, or to decline the question.

Responses were aggregated into broad ethnic groups for the purposes of this study – Māori, Pacific, Asian and European/other. Responses of ‘New Zealander’ (479 children (8.5%)) were included in the European/other group. None of the methods described below account for multiple ethnicities within an ethnic group.

Self-prioritised ethnicity was defined as the response to the prioritised ethnicity question above. If two ethnicities from different ethnic groups were indicated, then for the purposes of this study only, these responses were prioritised in the order Māori, Pacific, Asian and European/other. This resulted in a single ethnic group variable with four non-overlapping levels. ID hospitalisations were compared between these groups and also with those unable to prioritise a single ethnic group excluded. The European/other group was used as the comparator in these self-prioritised ethnicity analyses.

Total response ethnicity was defined as the response to the total response ethnicity question above, with membership of each ethnic group recorded separately. This resulted in four overlapping binary ethnic group variables. In unadjusted analyses, ID hospitalisations for a given ethnic group were compared with the combined pool of children not in that group, e.g. Māori children were compared with all non-Māori children. All four groups were included in the multivariable analyses. As this approach meant there was no single baseline for comparison, analyses were repeated using the non-overlapping ‘European/other only’ group as a comparator.

Single-combined ethnicity was also defined using responses to the total response ethnicity question, with individuals assigned to a single or combination ethnic group matching their combination of ethnicities. This resulted in a single ethnic group variable with 15 non-overlapping levels. As several combination groups included few individuals, analyses were also presented using a modified approach focussing on Māori and Pacific children, in which mixed ethnicities were aggregated into the following groups: Māori + other non-Pacific ethnicity, Pacific + other non-Māori ethnicity, Māori + Pacific ± other ethnicity, and European/other + Asian. The ‘European/other only’ ethnic group was used as the comparator in both analyses.

### Socio-economic variables

Household income was queried directly and primary caregivers were able to respond with a weekly, fortnightly, four-weekly, monthly or annual amount, before or after tax, from which annual income was calculated. Income brackets were offered to participants unable to answer, again before or after tax. NZ has a system of progressive marginal tax rates applied to individual earners, and tax credits which are available for lower income families. For post-tax amounts, it was not possible to calculate the corresponding pre-tax amount as household income may have been spread over several earners, and eligibility for tax credits was not recorded.

The NZDep is a census-derived, area-level measure of deprivation determined for the household using the usual address of the child [8]. The NZDep2013 was calculated from 2013 national census responses covering aspects of deprivation including internet access, receipt of government benefits, household income, employment status, educational qualifications, home ownership, family structure, household crowding and access to a car. An ordinal scale from 1 (least deprived 10%) to 10 (most deprived 10%) is calculated for each census meshblock – an area containing on average 81 people.

The NZiDep is an individualised index of deprivation, calculated for the primary caregiver from responses to eight interview questions regarding unemployment, receipt of government benefits and community charity, and the need to economise on food, heating and footwear [9]. The interview questions, and the standard NZiDep questionnaire, are included in Supplementary Table S1. A score from 1 (no responses suggesting deprivation) to 5 (5–8 responses suggesting deprivation) is calculated. Comparisons of ID hospitalisations were performed using the score and a binary variable comparing those scoring 1 and 2 with those scoring 3, 4 and 5.

### Statistical analyses

Analyses were performed using SAS version 9.4 software (SAS Institute, Cary, NC, USA). Proportions hospitalised for an ID and risk differences between ethnicity methods were calculated. Unadjusted analyses were presented using relative risks, 95% confidence intervals and *p*-values derived from the *χ*^2^ or Fisher's exact tests. Multivariable analyses were performed using log-binomial regression and were likewise presented as relative risks with 95% confidence intervals. Log-binomial regression was performed in preference to logistic regression as the outcome used was not rare. Multivariable models were first built using only the multiple levels of the variable of interest, then repeated with ethnicity models corrected for socio-economic deprivation using the NZDep2013 quintiles and socio-economic models corrected for ethnicity using self-prioritised ethnicity.

## Results

### The effect of ethnicity measures on risk of hospitalisation for an ID

A total of 5602 children had results available from both the 4½-year interview and linked hospitalisation data from the NMDS. A single self-prioritised ethnicity was provided for 4991 (89.1%) children, while two were provided for 528 (9.4%) children, 133 (2.4%) within one broad ethnic group and 395 (7.1%) across two ethnic groups. The caregivers of 83 (1.5%) children declined to prioritise an ethnicity. A single total-response ethnicity was identified for 3062 (54.7%) children with the remainder having multiple ethnicities identified. As some of these multiple ethnicities fell within the same broad ethnic group, 3880 (69.3%) children had a single total response ethnic group. A majority of children in each total response ethnic group were self-prioritised to the corresponding group, as shown in Supplementary Table S2.

Table 1 shows the relative risk of hospitalisation for an ID for each ethnic group by method of assigning ethnicity. Māori and Pacific children had a greater risk of hospitalisation for an ID across all methods of ascribing ethnicity and this was only partially reduced by correction for socio-economic deprivation. Relative risk estimates were higher for Māori and Pacific children when self-prioritised ethnicity was used compared with total-response ethnicity. When no fixed comparator group was used, European/other total response ethnicity was associated with a lower risk of hospitalisation for an ID. Using single-combined ethnicity, the larger groups containing Māori or Pacific children showed an increased risk for hospitalisation with an ID, as did the ‘Asian only’ group. No significant associations could be identified for the smallest combined ethnicity groups containing <100 children. After aggregation, a significant association with hospitalisation for an ID was apparent for all single or combination groups containing children with Māori or Pacific ethnicity. Higher relative risk estimates were seen for the Māori-only, Pacific-only and Māori + Pacific (±other) groups than for the mixed Māori + non-Pacific and Pacific + non-Māori groups.

**Table 1.** The effect of different methodologies of ascribing child ethnicity on relative risk for hospitalisation of an infectious disease (ID) in the first 5 years of life amongst 5602 children enrolled in the *Growing Up in New Zealand* longitudinal cohort study

		ID hospitalisation		Unadjusted		Multivariable^a^		Multivariable, adjusted^a^	
	*n* (%)	Yes	No	relative risk (95% CI)	*P*-value	relative risk (95% CI)	*P*-value	relative risk (95% CI)	*P*-value
Self-prioritised ethnicity									
European/Other	3266 (58.3)	664 (20.3)	2602 (79.7)	Reference		Reference		Reference	
Māori	858 (15.3)	288 (33.6)	570 (66.4)	1.65 (1.47–1.85)	<0.0001	1.65 (1.47–1.85)	<0.0001	1.48 (1.30–1.67)	<0.0001
Pacific	777 (13.9)	332 (42.7)	445 (57.3)	2.10 (1.89–2.34)	<0.0001	2.10 (1.89–2.33)	<0.0001	1.79 (1.58–2.03)	<0.0001
Asian	701 (12.5)	169 (24.1)	532 (75.9)	1.19 (1.02–1.37)	0.03	1.19 (1.02–1.37)	0.03	1.14 (0.97–1.31)	0.10
Self-prioritised ethnicity, excl. dual/non-responses									
European/Other	3177 (63.7)	645 (20.3)	2532 (79.7)	Reference		Reference		Reference	
Māori	538 (10.8)	180 (33.5)	358 (66.5)	1.65 (1.44–1.89)	<0.0001	1.65 (1.43–1.89)	<0.0001	1.46 (1.25–1.68)	<0.0001
Pacific	655 (13.1)	288 (44.0)	367 (56.0)	2.17 (1.94–2.42)	<0.0001	2.17 (1.94–2.42)	<0.0001	1.81 (1.59–2.07)	<0.0001
Asian	621 (12.4)	152 (24.5)	469 (75.5)	1.21 (1.03–1.41)	0.02	1.21 (1.03–1.40)	0.02	1.15 (0.98–1.34)	0.08
Total response ethnicity									
European/Other	4292 (76.6)	978 (22.8)	3314 (77.2)	0.63 (0.57–0.69)	<0.0001	0.72 (0.64–0.81)	<0.0001	0.78 (0.69–0.88)	<0.0001
Māori	1379 (24.6)	424 (30.8)	955 (69.3)	1.26 (1.15–1.39)	<0.0001	1.20 (1.09–1.31)	0.0002	1.14 (1.04–1.26)	0.007
Pacific	1117 (19.9)	444 (39.8)	673 (60.3)	1.77 (1.61–1.93)	<0.0001	1.48 (1.32–1.65)	<0.0001	1.36 (1.21–1.52)	<0.0001
Asian	823 (14.7)	206 (25.0)	617 (75.0)	0.96 (0.84–1.09)	0.52	0.91 (0.79–1.05)	0.23	0.93 (0.80–1.07)	0.34
Total response ethnicity, fixed comparator group									
European/other ONLY	2738 (48.9)	549 (20.1)	2189 (79.9)	Reference		Reference		Reference	
Māori	1379 (24.6)	424 (30.8)	955 (69.3)	1.53 (1.38–1.71)	<0.0001	1.19 (1.08–1.31)	0.0003	1.13 (1.02–1.24)	0.02
Pacific	1117 (19.9)	444 (39.8)	673 (60.3)	1.98 (1.79–2.20)	<0.0001	1.74 (1.59–1.91)	<0.0001	1.50 (1.35–1.66)	<0.0001
Asian	823 (14.7)	206 (25.0)	617 (75.0)	1.25 (1.09–1.44)	0.002	1.08 (0.95–1.22)	0.24	1.05 (0.92–1.19)	0.46
Single-combined ethnicity									
European/other only	2738 (48.9)	549 (20.1)	2189 (79.9)	Reference		Reference		Reference	
Māori only	157 (2.8)	64 (40.8)	93 (59.2)	2.03 (1.66–2.49)	<0.0001	2.03 (1.64–2.46)	<0.0001	1.76 (1.40–2.15)	<0.0001
Pacific only	476 (8.5)	216 (45.4)	260 (54.6)	2.26 (2.00–2.56)	<0.0001	2.26 (2.00–2.56)	<0.0001	1.89 (1.63–2.19)	<0.0001
Asian only	509 (9.1)	132 (25.9)	377 (74.1)	1.29 (1.10–1.53)	0.003	1.29 (1.09–1.52)	0.002	1.23 (1.04–1.45)	0.02
European + Māori	830 (14.8)	212 (25.5)	618 (74.5)	1.27 (1.11–1.46)	0.0007	1.27 (1.11–1.46)	0.0006	1.19 (1.03–1.36)	0.02
European + Pacific	247 (4.4)	74 (30.0)	173 (70.0)	1.49 (1.22–1.83)	0.0002	1.49 (1.21–1.82)	0.0001	1.37 (1.10–1.67)	0.003
European + Asian	211 (3.8)	43 (20.4)	168 (79.6)	1.02 (0.77–1.34)	0.91	1.02 (0.76–1.32)	0.91	0.99 (0.74–1.29)	0.95
Māori + Pacific	133 (2.4)	50 (37.6)	83 (62.4)	1.87 (1.49–2.36)	<0.0001	1.88 (1.46–2.33)	<0.0001	1.59 (1.23–2.00)	0.0002
Māori + Asian	<10 (<0.2)	<10 (42.9)	<10 (57.1)	2.14 (0.91–5.04)	0.15	2.14 (0.64–3.89)	0.08	1.95 (0.59–3.52)	0.13
Pacific + Asian	22 (0.4)	<10 (36.4)	14 (63.6)	1.81 (1.04–3.17)	0.06	1.81 (0.92–2.87)	0.04	1.57 (0.80–2.49)	0.11
European + Māori + Pacific	197 (3.5)	82 (41.6)	115 (58.4)	2.08 (1.73–2.49)	<0.0001	2.08 (1.72–2.47)	<0.0001	1.84 (1.51–2.21)	<0.0001
European + Māori + Asian	32 (0.6)	<10 (18.8)	26 (81.3)	0.94 (0.45–1.93)	0.85	0.94 (0.39–1.73)	0.86	0.88 (0.37–1.62)	0.72
European + Pacific + Asian	19 (0.3)	<10 (36.8)	12 (63.2)	1.84 (1.02–3.33)	0.07	1.84 (0.89–2.98)	0.04	1.64 (0.79–2.65)	0.10
Māori + Pacific + Asian	<10 (<0.2)	<10 (40.0)	<10 (60.0)	1.99 (0.68–5.85)	0.26	2.00 (0.40–4.02)	0.21	1.61 (0.32–3.28)	0.39
European + Māori + Pacific + Asian	18 (0.3)	<10 (27.8)	13 (72.2)	1.39 (0.66–2.93)	0.38	1.39 (0.55–2.53)	0.39	1.26 (0.50–2.30)	0.54
Single-combined ethnicity, aggregated combinations									
European/other only	2738 (48.9)	549 (20.1)	2189 (80.0)	Reference		Reference		Reference	
Māori only	157 (2.8)	64 (40.8)	93 (59.2)	2.03 (1.66–2.49)	<0.0001	2.03 (1.64–2.46)	<0.0001	1.76 (1.40–2.15)	<0.0001
Pacific only	476 (8.5)	216 (45.4)	260 (54.6)	2.26 (2.00–2.56)	<0.0001	2.26 (2.00–2.56)	<0.0001	1.89 (1.63–2.19)	<0.0001
Asian only	509 (9.1)	132 (25.9)	377 (74.1)	1.29 (1.10–1.53)	0.003	1.29 (1.09–1.52)	0.002	1.23 (1.04–1.45)	0.02
Māori + non-Pacific	869 (15.5)	221 (25.4)	648 (74.6)	1.27 (1.11–1.45)	0.0007	1.27 (1.10–1.45)	0.0006	1.18 (1.03–1.36)	0.02
Pacific + non-Māori	288 (5.1)	89 (30.9)	199 (69.1)	1.54 (1.28–1.86)	<0.0001	1.54 (1.27–1.85)	<0.0001	1.40 (1.15–1.69)	0.0006
Māori + Pacific ± others	353 (6.3)	139 (39.4)	214 (60.6)	1.96 (1.69–2.28)	<0.0001	1.96 (1.68–2.27)	<0.0001	1.71 (1.45–2.00)	<0.0001
European + Asian	211 (3.8)	43 (20.4)	168 (79.6)	1.02 (0.77–1.34)	0.91	1.02 (0.76–1.32)	0.91	0.99 (0.74–1.29)	0.95

a The multivariable model was first constructed with only the multiple levels of the relevant ethnicity variable. The ‘adjusted’ column represents the same model after adjustment for socio-economic status using the NZDep2013.

When risk differences between corresponding ethnic groups using different methods were calculated (Table 2), a slightly higher risk for European/other children and a non-significant trend towards lower risk for Māori and Pacific children were seen when using total response compared with self-prioritised. Self-prioritised Māori ethnicity appeared to overestimate the risk for mixed Māori + non-Pacific children defined using aggregated single-combined ethnicity. Likewise, Pacific self-prioritised ethnicity appeared to overestimate the risk for mixed Pacific + non-Māori children. These differences were further accentuated when comparing total response with single-combined ethnicity. In addition, total response Māori ethnicity was found to underestimate the risk for Māori-only, and mixed Māori + Pacific children, while total response Pacific ethnicity underestimated the risk for Pacific-only children.

**Table 2.** Comparison of the proportion of children from corresponding ethnic groups hospitalised for an infectious disease (ID) using different methodologies of ascribing child ethnicity

	Percentage (95% CI) hospitalised for an ID by methodology of ascribing ethnicity				
Ethnic group method comparison	Self-prioritised	Total response	Single-combined	Risk difference	*P*-value
Self-prioritised *vs*. total response					
SP European/other *vs*. TR European/other	20.3 (19.0–21.7)	22.8 (21.5–24)		−2.5 (−4.3 to −0.6)	0.01
SP Māori *vs*. TR Māori	33.6 (30.4–36.7)	30.8 (28.3–33.2)		2.8 (−1.2 to 6.8)	0.16
SP Pacific *vs*. TR Pacific	42.7 (39.3–46.2)	39.8 (36.9–42.6)		3.0 (−1.5 to 7.5)	0.19
SP Asian *vs*. TR Asian	24.1 (20.9–27.3)	25.0 (22.1–28.0)		−0.9 (−5.3 to 3.4)	0.68
Self-prioritised *vs*. single-combined					
SP European/other *vs*. SC European/other only	20.3 (19.0–21.7)		20.1 (18.6–21.6)	−0.3 (−2.3 to 1.8)	0.79
SP European/other *vs*. SC European + Asian	20.3 (19.0–21.7)		20.4 (14.9–25.8)	0.1 (−5.6 to 5.7)	0.99
SP Māori *vs*. SC Māori only	33.6 (30.4–36.7)		40.8 (33.1–48.5)	7.2 (−1.1 to 15.5)	0.08
SP Māori *vs*. SC Māori + non-Pacific	33.6 (30.4–36.7)		25.4 (22.5–28.3)	−8.1 (−12.4 to −3.9)	0.0002
SP Māori *vs*. SC Māori + Pacific ± others	33.6 (30.4–36.7)		39.4 (34.3–44.5)	5.8 (−0.2 to 11.8)	0.05
SP Pacific *vs*. SC Pacific only	42.7 (39.3–46.2)		45.4 (40.9–49.9)	2.7 (−3.0 to 8.3)	0.36
SP Pacific *vs*. SC Pacific + non-Māori	42.7 (39.3–46.2)		30.9 (25.6–36.2)	−11.8 (−18.2 to −5.5)	0.0005
SP Pacific *vs*. SC Māori + Pacific ± others	42.7 (39.3–46.2)		39.4 (34.3–44.5)	−3.4 (−9.5 to 2.8)	0.29
SP Asian *vs*. SC Asian only	24.1 (20.9–27.3)		25.9 (22.1–29.7)	1.8 (−3.1 to 6.8)	0.47
SP Asian *vs*. SC European + Asian	24.1 (20.9–27.3)		20.4 (14.9–25.8)	−3.7 (−10 to 2.6)	0.26
Total response *vs*. single-combined					
TR European/other *vs*. SC European/other only		22.8 (21.5–24)	20.1 (18.6–21.6)	−2.7 (−4.7 to −0.8)	0.007
TR European/other *vs*. SC European + Asian		22.8 (21.5–24)	20.4 (14.9–25.8)	−2.4 (−8.0 to 3.2)	0.41
TR Māori *vs*. SC Māori only		30.8 (28.3–33.2)	40.8 (33.1–48.5)	10.0 (2.0–18.1)	0.01
TR Māori *vs*. SC Māori + non-Pacific		30.8 (28.3–33.2)	25.4 (22.5–28.3)	−5.3 (−9.1 to −1.5)	0.007
TR Māori *vs*. SC Māori + Pacific ± others		30.8 (28.3–33.2)	39.4 (34.3–44.5)	8.6 (3.0–14.3)	0.002
TR Pacific *vs*. SC Pacific only		39.8 (36.9–42.6)	45.4 (40.9–49.9)	5.6 (0.3–10.9)	0.04
TR Pacific *vs*. SC Pacific + non-Māori		39.8 (36.9–42.6)	30.9 (25.6–36.2)	−8.9 (−14.9 to −2.8)	0.006
TR Pacific *vs*. SC Māori + Pacific ± others		39.8 (36.9–42.6)	39.4 (34.3–44.5)	−0.4 (−6.2 to 5.5)	0.9
TR Asian *vs*. SC Asian only		25.0 (22.1–28.0)	25.9 (22.1–29.7)	0.9 (−3.9 to 5.7)	0.71
TR Asian *vs*. SC European + Asian		25.0 (22.1–28.0)	20.4 (14.9–25.8)	−4.7 (−10.8 to 1.5)	0.16

SP, self-prioritised ethnicity; TR, total response ethnicity; SC, single-combined ethnicity.

### The effect of socio-economic status measures of risk of hospitalisation for an ID

Pre-tax household income was provided by 3833 (68.4%) primary caregivers and post-tax income by 1165 (20.8%). Household income was not provided by 604 (10.8%) primary caregivers. These data were not missing at random – 5.0% of those in the least deprived NZDep2013 quintile and 21.5% of those in the most deprived NZDep2013 quintile had missing household income data (*p* < 0.0001). In addition, post-tax income was provided by 15.0% of those in the least deprived quintile and by 35.0% of those in the most deprived quintile (*p* < 0.0001). Due to the risk of bias and the inability to correlate pre- and post-tax income accurately, further analyses were not performed using household income.

While the NZDep2013 resulted in quintiles of similar size (range 952–1373), the NZiDep gave 55.1% of primary caregivers a score of 1. Both systems demonstrated a social gradient in relative risk of hospitalisation for an ID that was maintained after correction for ethnicity, as shown in Table 3. The full range of NZiDep scores was seen within each NZDep2013 quintile but there was a strong relationship between increasing NZDep2013 quintile and increasing NZiDep score, whether used directly or in a binary form (Supplementary Table S3).

**Table 3.** The effect of different methodologies of ascribing socio-economic status on relative risk of hospitalisation for an infectious disease (ID) in the first 5years of life amongst 5602 children enrolled in the *Growing Up in New Zealand* longitudinal cohort study

		ID hospitalisation		Unadjusted		Multivariable^a^		Multivariable, adjusted^a^	
	*n* (%)	Yes	No	relative risk (95% CI)	*P*–value	relative risk (95% CI)	*P*–value	relative risk (95% CI)	*P*–value
NZDep2013 deciles									
1–2 (least deprived)	1191 (21.3)	231 (19.4)	960 (80.6)	Reference		Reference		Reference	
3–4	1087 (19.4)	221 (20.3)	866 (79.7)	1.05 (0.89–1.24)	0.58	1.05 (0.89–1.24)	0.58	1.01 (0.86–1.19)	0.90
5–6	999 (17.8)	241 (24.1)	758 (75.9)	1.24 (1.06–1.46)	0.007	1.24 (1.06–1.46)	0.007	1.16 (0.99–1.36)	0.07
7–8	952 (17.0)	261 (27.4)	691 (72.6)	1.41 (1.21–1.65)	<0.0001	1.41 (1.21–1.65)	<0.0001	1.21 (1.03–1.42)	0.02
9–10 (most deprived)	1373 (24.5)	499 (36.3)	874 (63.7)	1.87 (1.64–2.15)	<0.0001	1.87 (1.64–2.15)	<0.0001	1.38 (1.18–1.60)	<0.0001
NZiDep score									
1	3071 (55.1)	668 (21.8)	2403 (78.3)	Reference		Reference		Reference	
2	1161 (20.8)	295 (25.4)	866 (74.6)	1.17 (1.04–1.32)	0.01	1.17 (1.04–1.31)	0.01	1.08 (0.95–1.21)	0.22
3	531 (9.5)	171 (32.2)	360 (67.8)	1.48 (1.29–1.70)	<0.0001	1.48 (1.28–1.70)	<0.0001	1.29 (1.12–1.48)	0.0004
4	500 (9.0)	186 (37.2)	314 (62.8)	1.71 (1.50–1.95)	<0.0001	1.71 (1.49–1.95)	<0.0001	1.37 (1.19–1.57)	<0.0001
5	309 (5.6)	119 (38.5)	190 (61.5)	1.77 (1.51–2.07)	<0.0001	1.77 (1.51–2.06)	<0.0001	1.30 (1.10–1.52)	0.002
NZiDep score, binary									
1–2	4232 (76.0)	963 (22.8)	3269 (77.2)	Reference		Reference		Reference	
3–5	1340 (24.1)	476 (35.5)	864 (64.5)	1.56 (1.43–1.71)	<0.0001	1.56 (1.42–1.71)	<0.0001	1.29 (1.17–1.42)	<0.0001

a The multivariable model was first constructed with only the multiple levels of the relevant socio-economic variable. The ‘adjusted’ column represents the same model after adjustment for ethnicity using the self-prioritised ethnic group method.

### Relationship between ethnicity and socio-economic status

Examination of socio-economic status within ethnic groups (Supplementary Tables S4.1 and S4.2) showed that Māori and Pacific children were more likely to live in socio-economically deprived households than Asian and European/other children. When Māori or Pacific ethnicity was defined using total response, the frequency of socio-economic deprivation was lower than when self-prioritisation was used. When aggregated single-combined ethnicity was used, a greater proportion of Māori only, Pacific only and Māori + Pacific (±other) children lived in socio-economically deprived households than did Māori + non-Pacific or Pacific + non-Māori children, while the European/other, Asian and European/other + Asian groups had the lowest proportions living in socio-economically deprived households.

When the effect of socio-economic deprivation on hospitalisation with an ID was examined within ethnic groups (Supplementary Tables S5.1 and S5.2), there was a general trend towards an association between increased deprivation and increased rates of hospitalisation. Ethnic subgroup size was important as the relationship was more likely to reach statistical significance when larger, total response ethnic groups were used, and when the NZiDep was used in a binary form.

## Discussion

This study has demonstrated several methods of ascribing child ethnicity and socio-economic status. Regardless of methodology, Māori and Pacific children, and children in the most socio-economically deprived households, had a higher risk of hospitalisation for an ID. However, the magnitude of these effects varied between methods. The GUiNZ cohort is large and includes representative proportions of Māori, Pacific, and socio-economically deprived families [10, 12]. In studies with lower statistical power, due to a smaller sample size or under-representation of disadvantaged population subgroups, the differences between methodologies are likely to be more important. There are also philosophical differences between methodologies which might make one method more appropriate than another in certain settings.

Ethnicity can be an area of significant individual and societal sensitivity, so the use of ethnicity data in research must be managed respectfully. Ethnicity is a self-determined construct deeply enmeshed with related aspects of identity including genetic or geographic ancestry, race, nationality, physical features, shared history, language or religion, and how society identifies the individual [6]. The borders of ethnic categories are indistinct, and subject to local political considerations making international comparisons difficult. While the relationship between ethnicity and ancestry leads to a small degree of genetic variation between ethnic groups [15], this is minor compared with within-group variation, meaning ethnicity functions more as a marker of risk due to social factors than as a risk factor in itself [16, 17]. Despite these complexities, ethnicity remains an important variable in epidemiological studies which seek to document disparities in health outcomes and to guide health resource allocation [17, 18]. However, many research articles compare outcomes by ethnic group without defining how ethnicity was determined. From a participant perspective, an ideal measure of ethnicity should be self-identified and capable of recording multiple ethnicities and the relative or equal importance of each. From a research perspective, the measure should remain simple to analyse and maintain subgroup size and statistical power.

External prioritisation to a single ethnicity, generally in the order Māori, Pacific, Asian, other non-European, then European, has fallen out of favour in NZ as it does not respect self-identification and fails to account for mixed ethnicities [7]. In addition, external prioritisation maximises the size of the higher priority groups by including individuals who may share relatively little of the ethnic identity and social disadvantage common to that group. Therefore, it may underestimate the burden of excess morbidity experienced by individuals who would prioritise themselves to that ethnic group.

The methods demonstrated in this study were all self-identified but differed in the way they treated multiple ethnicities, with consequences for analysis and interpretation. Self-prioritisation did not allow multiple ethnicities and yielded a single non-overlapping ethnic group per participant. As individuals were assigned to the group their caregiver felt they identified with most, dilution of social disadvantage was reduced – this likely explains the slightly (though non-significantly) greater risk estimates seen when compared with total response. However, 11% of the cohort as a whole and 25% of total response Māori children were unable to be prioritised; caregivers either chose two ethnicities or declined to answer. Excluding these individuals had only a small effect on risk estimates but should not be seen as an appropriate response to this problem. Ethnic diversity within the Māori cohort, a parental preference for the transference of Māori ethnicity, and the concept of the indivisibility of whakapapa (genealogy or descent, meaning that a person of Māori descent is regarded as Māori regardless of the proportion of their Māori ancestry), likely explain why caregivers of Māori children were particularly disinclined to prioritise a single ethnicity [19–21].

Total response ethnicity allowed caregivers to identify multiple ethnicities simultaneously but this overlap complicated analyses as no single comparator group was available. Philosophically, this lack of a comparator group has the advantage of avoiding the ethnocentric assumption that the comparator group has the desired level of the outcome [22]. This benefit is lost if a residual non-overlapping group (‘European/other only’ in this study) is used as a fixed comparator. Dilution of social disadvantage can be a problem where overlaps are large, and can be further compounded by inclusion of other disadvantaged groups in the pooled comparator.

Single-combined ethnicity also allowed multiple ethnicities but without overlap between groups. Multiple combination groups were identified, some of which had few participants with consequent loss of power and difficulty presenting results coherently. Use of finer measures of ethnicity than the broad groups used in this study would exacerbate this effect further. Aggregating the smaller combination groups overcame this issue at the expense of loss of detail. A particular strength of the single-combined method was the ability to demonstrate the diversity of risk within the Māori and Pacific groups, as defined using self-prioritisation or total response, showing that these groups are not homogeneous.

Comparison of measures of socio-economic status also identified several issues. This study compared household income with validated census-derived and survey-derived measures of socio-economic deprivation which are commonly used in NZ. Household income proved problematic as a measure of financial resources. Previous research has documented that responses to questions regarding income are frequently incomplete, inaccurate or declined [23]. Reasons for this can be informational, computational or motivational [24]. While both under- and over-reporting may occur [24], we found non-response to be most frequent amongst participants living in more deprived areas, creating a significant risk of bias if those with missing data were excluded. Participants living in more deprived areas were also more likely to provide post-tax income, compounding the risk of bias if only participants with pre-tax income were considered. As an additional limitation, the entire income amount may not necessarily be available as money may be used to pay interest on debt or may be diverted by charitable giving, particularly to religious institutions, or remittances to relatives overseas.

The NZDep2013 [8] and NZiDep [9, 25] performed similarly well in differentiating risk of hospitalisation for an ID across the socio-economic gradient, despite having different underlying methodologies. The NZDep is updated after each national census (NZDep2013 being the update following the 2013 census) and made publically available, including in atlas form [26], so it has the advantage of a high level of completeness if the residential address is known. Similar census-derived indices of deprivation have been used in the UK [27] and in limited areas in the USA [28], and can be used to determine health resource allocation as well as research [29]. There are a number of potential pitfalls with using the NZDep. First, the census questions used to derive the NZDep cover a range of social determinants of health. If similar variables, such as household crowding, are included with the NZDep in multivariable analyses, this creates a risk of multicollinearity. Second, deprivation is not homogeneous within NZDep deciles, therefore making assumptions about individuals within a given decile is a form of ecological fallacy. Comparison of NZiDep scores within NZDep2013 deciles showed that while high NZiDep scores were rare in the least deprived deciles, NZiDep scores of 1 or 2 were still common in the most deprived deciles, consistent with previous research in NZ [30].

The NZiDep required an additional eight questionnaire items but proved acceptable to participants with only 0.5% declining to answer one or more. While these questions covered employment and benefits, they did not cover other social determinants of health such as crowding, meaning that these variables could also have been included in multivariable analyses. As individualised data were used, albeit for the caregiver rather than the child, the NZiDep largely avoided the ecological fallacy concerns affecting the NZDep. The proportion of the cohort with NZiDep scores of 3, 4 or 5 was small, leading to reduced statistical power. Creating a binary variable by comparing those scoring 3, 4 or 5 with those scoring 1 or 2 preserved the size of the more deprived subgroup and appeared to work well. This approach would require further validation before widespread use. The responses to specific NZiDep questions may also be of interest in their own right, and may highlight areas of hardship, such as food poverty, for policy intervention.

This study had some limitations. The use of broad ethnic groups was artificial. Only the Māori group represented an ethnicity many people would identify with. This approach obscured within-group mixed ethnicities, such as Tongan and Samoan, or Chinese and Korean. However, it is similar to Statistics New Zealand's use of ‘level 1’ ethnicity, and to the use of ethnicity in much international health research, and maintains statistical power. This study used ethnicity data obtained from the primary caregiver, most commonly the mother, and not their partner. Parents may have differed in their interpretation or weighting of their child's ethnicity [20]. The intergenerational transmission of ethnicity is not straightforward, especially in inter-ethnic parental relationships or where one or both parents identify as mixed ethnicity themselves [19]. The frequent use of the ‘New Zealander’ response was problematic. We included these children in the European/other category, but in reality, the group is heterogeneous. Similar issues have been faced in interpreting national census results [31]. The NZiDep questions were not all taken verbatim from the validated questionnaire, so items describing employment status and receipt of benefits had to be recoded from alternative survey questions which closely approximated the NZiDep items. A high proportion (17.3%) of primary caregivers were not working due to childcare or family commitments. This scored a ‘no’ on the unemployment item and may have led to an underestimate of deprivation in some of these families. Neither of the socio-economic deprivation indices directly described material deprivation at the level of the individual child. GUINZ has collected numerous additional variables describing social determinants of health including household crowding, housing quality, tobacco smoke exposure, healthcare access, exposure to racism and others, all of which were intentionally excluded from this demonstration. GUiNZ is a longitudinal study incorporating data from multiple data collection waves, however the current study used only cross-sectional data at 4½ years of child age. Further research is required to investigate longitudinal changes in child ethnic identity and socio-economic mobility.

In summary, this study has demonstrated multiple methods of ascribing ethnicity and socio-economic deprivation in a large and diverse child cohort. Self-prioritised, total response and single combined ethnicity were all usable. Self-prioritisation was simplest to analyse, but 10% of participants could not be prioritised to a single ethnic group. Total response was complicated by overlap between groups and, in an unmodified form, did not allow a single baseline for comparison. Single-combined created a number of small ethnic groups with loss of statistical power, but aggregation overcame this. Single-combined ethnicity revealed diversity of risk within the broader Māori and Pacific groups and for this reason, and with mixed ethnicity becoming increasingly common, the single-combined method should be preferred where sample size and data structure allow it. Household income was affected by non-random missing data and the inability to combine pre-tax and post-tax income, both factors contributing to a risk of bias. Both NZDep2013 and the NZiDep were effective in differentiating risk between high and low levels of deprivation. The NZDep2013 requires caution with regards to the ecological fallacy and is most appropriate to studies which do not include social determinants of health which overlap with the census items used to derive the index. The NZiDep avoids these issues but requires a large sample size as a relatively small proportion of people are identified as having high deprivation scores. The questions from which the NZiDep is derived may be of interest in their own right.

## Supplementary material

For supplementary material accompanying this paper visit https://doi.org/10.1017/S0950268818002935. click here to view supplementary material
