# Supplementary material for: How differing methods of ascribing ethnicity and socio-economic status affect risk estimates for hospitalisation with infectious disease
Source: Epidemiol Infect. 2018 Nov 13;147:e40. doi: 10.1017/S0950268818002935 (PMC6518588; doi:10.1017/S0950268818002935)
Supplement: Supplementary file 1 [file S0950268818002935sup001.docx]

**Epidemiology and Infection**

**Title:** How differing methods of ascribing ethnicity and socioeconomic status affect risk estimates for hospitalization with infectious disease.

**Authors:** Hobbs MR. Atatoa Carr P. Fa’alili-Fidow J. Pillai A. Morton SMB. Grant CC.

**Supplementary material**

**Supplementary Table S1:** Standard NZiDep questions, *Growing Up in New Zealand* (GUINZ) questionnaire items used to derive NZiDep, and participant response rates from the primary caregivers of 5602 cohort children.

| NZiDep 2014 question | GUINZ question | Responses | | |
| --- | --- | --- | --- | --- |
|  |  | **Yes (%)** | **No (%)** | **Missing (%)** |
| 1 – In the last 12 months have you personally been forced to buy cheaper food so that you could pay for other things you needed? | Unchanged | 1994 (35.5) | 3598 (64.2) | 10 (0.2) |
| 2 – In the last 12 months, have you been out of paid work at any time for more than one month? | Recoded from responses to multiple questions. See footnote. | 555 (9.9) | 5036 (89.9) | 11 (0.2) |
| 3 – In the 12 months ending today did you yourself receive payments from any of these three benefits: Jobseeker Support, Sole Parent Support or Supported Living Payment? | Which of the following are current sources of income for your household? Recoded from affirmative responses for: Jobseeker Support; Sole Parent Support; Supported Living Payment. | 611 (10.9) | 4984 (89.0) | <10 (0.1) |
| 4 – In the last 12 months have you personally put up with feeling cold to save heating costs? | Unchanged | 787 (14.0) | 4809 (85.8) | <10 (0.1) |
| 5 – In the last 12 months have you personally made use of special food grants or food banks because you did not have enough money for food? | Unchanged | 506 (9.0) | 5092 (90.9) | <10 (0.1) |
| 6 – In the last 12 months have you personally continued wearing shoes with holes because you could not afford replacements? | Unchanged | 541 (9.7) | 5057 (90.3) | <10 (0.1) |
| 7 – In the last 12 months have you personally gone without fresh fruit and vegetables, often, so that you could pay for other things you needed? | Unchanged | 574 (10.2) | 5022 (89.6) | <10 (0.1) |
| 8 – In the last 12 months have you personally received help in the form of clothes or money from a community organisation (like the Salvation Army)? | Unchanged | 256 (4.6) | 5341 (95.3) | <10 (0.1) |

**Supplementary Table 1 Footnote**: The responses from multiple questions asked about primary caregiver employment status were recoded to derive a response to Question 2. These included:

1. Which of the following best describes your current situation in regard to paid work?
2. How long have you been, or how long were you, seeking work?
3. What is the MAIN reason you are not currently in paid work?

**Supplementary Table S2:** Frequency of self-prioritized ethnic groups within total response ethnic groups for 5602 children enrolled in the *Growing Up in New Zealand* longitudinal cohort study.

|  | Total response ethnicity | | | |
| --- | --- | --- | --- | --- |
|  | **European/Other** | **Māori** | **Pacific** | **Asian** |
| Total | 4292 (100.0) | 1379 (100.0) | 1117 (100.0) | 823 (100.0 ) |
|  |  |  |  |  |
| Self-prioritised ethnicity |  |  |  |  |
| European/Other | 3265 (76.1) | 426 (30.9) | 123 (11.0) | 94 (11.4) |
| Māori | 601 (14.0) | 858 (62.2) | 221 (19.8) | 35 (4.3) |
| Pacific | 239 (5.6) | 92 (6.7) | 767 (68.7) | 39 (4.7) |
| Asian | 187 (4.4) | <10 (<1.0) | <10 (<1.0) | 655 (79.6) |

**Supplementary Table S3:** Frequency of NZiDep score within NZDep2013 deciles for the primary caregivers of 5602 children enrolled in the *Growing Up in New Zealand* longitudinal cohort study.

|  | NZDep2013 Deciles | | | | |
| --- | --- | --- | --- | --- | --- |
|  | **1 to 2** | **3 to 4** | **5 to 6** | **7 to 8** | **9 to 10** |
| Total | 1187 (100.0) | 1085 (100.0) | 994 (100.0) | 944 (100.0) | 1362 (100.0) |
|  |  |  |  |  |  |
| NZiDep Score |  |  |  |  |  |
| 1 | 854 (71.95) | 739 (68.11) | 601 (60.46) | 465 (49.26) | 412 (30.25) |
| 2 | 221 (18.62) | 220 (20.28) | 206 (20.72) | 213 (22.56) | 301 (22.10) |
| 3 | 70 (5.90) | 65 (5.99) | 99 (9.96) | 109 (11.55) | 188 (13.80) |
| 4 | 33 (2.78) | 46 (4.24) | 62 (6.24) | 93 (9.85) | 266 (19.53) |
| 5 | <10 (<1.0) | 15 (1.38) | 26 (2.62) | 64 (6.78) | 195 (14.32) |
|  |  |  |  |  |  |
| NZiDep Score, binary |  |  |  |  |  |
| 1 to 2 | 1075 (90.56) | 959 (88.39) | 807 (81.19) | 678 (71.82) | 713 (52.35) |
| 3 to 5 | 112 (9.44) | 126 (11.61) | 187 (18.81) | 266 (28.18) | 649 (47.65) |

**Supplementary Table S4.1:** Distribution of socioeconomic status (NZDep2013 deciles and NZiDep scores) within self-prioritized and total response ethnic groups for 5602 children enrolled in the *Growing Up in New Zealand* longitudinal cohort study.

|  | Self-prioritised ethnicity, n (%) | | | | Total response ethnicity, n (%) | | | |
| --- | --- | --- | --- | --- | --- | --- | --- | --- |
|  | **Euro/Other** | **Māori** | **Pacific** | **Asian** | **Euro/Other** | **Māori** | **Pacific** | **Asian** |
| NZDep2013 deciles |  |  |  |  |  |  |  |  |
| 1 – 2 (least deprived) | 994 (30.4) | 71 (8.3) | 21 (2.7) | 105 (15.0) | 1110 (25.9) | 168 (12.2) | 45 (4.0) | 129 (15.7) |
| 3 – 4 | 782 (23.9) | 90 (10.5) | 49 (6.3) | 166 (23.7) | 931 (21.7) | 172 (12.5) | 96 (8.6) | 182 (22.1) |
| 5 – 6 | 674 (20.6) | 125 (14.6) | 65 (8.4) | 135 (19.3) | 844 (19.7) | 224 (16.2) | 113 (10.1) | 157 (19.1) |
| 7 – 8 | 475 (14.5) | 187 (21.8) | 135 (17.4) | 155 (22.1) | 700 (16.3) | 283 (20.5) | 201 (18.0) | 176 (21.4) |
| 9 – 10 (most deprived) | 341 (10.4) | 385 (44.9) | 507 (65.3) | 140 (20.0) | 707 (16.5) | 532 (38.6) | 662 (59.3) | 179 (21.8) |
|  |  |  |  |  |  |  |  |  |
| NZiDep score |  |  |  |  |  |  |  |  |
| 1 | 2074 (63.7) | 306 (36.0) | 216 (27.9) | 475 (68.7) | 2515 (58.8) | 540 (39.4) | 353 (31.8) | 524 (64.5) |
| 2 | 650 (20.0) | 199 (23.4) | 176 (22.8) | 136 (19.7) | 897 (21.0) | 317 (23.2) | 248 (22.3) | 170 (20.9) |
| 3 | 274 (8.4) | 118 (13.9) | 99 (12.8) | 40 (5.8) | 397 (9.3) | 191 (14.0) | 149 (13.4) | 54 (6.6) |
| 4 | 184 (5.7) | 137 (16.1) | 150 (19.4) | 29 (4.2) | 314 (7.3) | 198 (14.5) | 194 (17.5) | 45 (5.5) |
| 5 | 76 (2.3) | 90 (10.6) | 132 (17.1) | 11 (1.6) | 157 (3.7) | 123 (9.0) | 166 (15.0) | 20 (2.5) |
|  |  |  |  |  |  |  |  |  |
| NZiDep score, binary |  |  |  |  |  |  |  |  |
| 1 – 2 | 2724 (83.6) | 505 (59.4) | 392 (50.7) | 611 (88.4) | 3412 (79.7) | 857 (62.6) | 601 (54.1) | 694 (85.4) |
| 3 – 5 | 534 (16.4) | 345 (40.6) | 381 (49.3) | 80 (11.6) | 868 (20.3) | 512 (37.4) | 509 (45.9) | 119 (14.6) |

**Supplementary Table S4.2:** Socioeconomic status (NZDep2013 and NZiDep) within aggregated single-combined ethnic groups for 5602 children enrolled in the *Growing Up in New Zealand* longitudinal cohort study.

|  | Single-combined ethnic group, n (%) | | | | | | | |
| --- | --- | --- | --- | --- | --- | --- | --- | --- |
|  | **Euro/Other only** | **Māori only** | **Pacific only** | **Asian only** | **Māori + non-Pacific** | **Pacific + non-Māori** | **Māori + Pacific** | **Euro/Other + Asian** |
| NZDep2013 deciles |  |  |  |  |  |  |  |  |
| 1 – 2 (least deprived) | 873 (31.9) | <10 (5.1) | <10 (0.6) | 67 (13.2) | 145 (16.7) | 27 (9.4) | 15 (4.3) | 52 (24.6) |
| 3 – 4 | 679 (24.8) | 10 (6.4) | 15 (3.2) | 120 (23.6) | 134 (15.4) | 53 (18.4) | 28 (7.9) | 48 (22.8) |
| 5 – 6 | 562 (20.5) | 10 (6.4) | 28 (5.9) | 107 (21.0) | 173 (19.9) | 44 (15.3) | 41 (11.6) | 34 (16.1) |
| 7 – 8 | 390 (14.2) | 43 (27.4) | 72 (15.1) | 108 (21.2) | 163 (18.8) | 52 (18.1) | 77 (21.8) | 47 (22.3) |
| 9 – 10 (most deprived) | 234 (8.6) | 86 (54.8) | 358 (75.2) | 107 (21.0) | 254 (29.2) | 112 (38.9) | 192 (54.4) | 30 (14.2) |
|  |  |  |  |  |  |  |  |  |
| NZiDep score |  |  |  |  |  |  |  |  |
| 1 | 1804 (66.0) | 45 (29.0) | 115 (24.3) | 351 (70.3) | 381 (44.1) | 124 (43.2) | 114 (32.6) | 136 (64.5) |
| 2 | 531 (19.4) | 35 (22.6) | 101 (21.4) | 96 (19.2) | 207 (24.0) | 72 (25.1) | 75 (21.4) | 44 (20.9) |
| 3 | 204 (7.5) | 21 (13.6) | 59 (12.5) | 27 (5.4) | 115 (13.3) | 35 (12.2) | 55 (15.7) | 15 (7.1) |
| 4 | 142 (5.2) | 29 (18.7) | 104 (22.0) | 17 (3.4) | 106 (12.3) | 27 (9.4) | 63 (18.0) | 12 (5.7) |
| 5 | 51 (1.9) | 25 (16.1) | 94 (19.9) | <10 (1.6) | 55 (6.4) | 29 (10.1) | 43 (12.3) | <10 (1.9) |
|  |  |  |  |  |  |  |  |  |
| NZiDep score, binary |  |  |  |  |  |  |  |  |
| 1 – 2 | 2335 (85.5) | 80 (51.6) | 216 (45.7) | 447 (89.6) | 588 (68.1) | 196 (68.3) | 189 (54.0) | 180 (85.3) |
| 3 – 5 | 397 (14.5) | 75 (48.4) | 257 (54.3) | 52 (10.4) | 276 (31.9) | 91 (31.7) | 161 (46.0) | 31 (14.7) |

**Supplementary Table S5.1:** Percentage of 5602 children enrolled in the *Growing Up in New Zealand* longitudinal cohort study hospitalised for an infectious disease within self-prioritised and total response ethnic groups, by measure of socioeconomic status (NZDep2013 and NZiDep).

|  | Self-prioritised ethnic group | | | | | | | | Total response ethnic group | | | | | | | |
| --- | --- | --- | --- | --- | --- | --- | --- | --- | --- | --- | --- | --- | --- | --- | --- | --- |
|  | **Euro/Other** | | **Māori** | | **Pacific** | | **Asian** | | **Euro/Other** | | **Māori** | | **Pacific** | | **Asian** | |
|  | **%** | **P** | **%** | **P** | **%** | **P** | **%** | **P** | **%** | **P** | **%** | **P** | **%** | **P** | **%** | **P** |
| NZDep2013 |  |  |  |  |  |  |  |  |  |  |  |  |  |  |  |  |
| 1 – 2 | 18.7 | 0.07 | 23.9 | 0.07 | 38.1 | 0.11 | 19.1 | 0.03 | 19.4 | <0.0001 | 23.2 | 0.001 | 40.0 | 0.004 | 16.3 | 0.0005 |
| 3 – 4 | 19.1 |  | 25.6 |  | 40.8 |  | 17.5 |  | 20.0 |  | 23.3 |  | 29.2 |  | 17.6 |  |
| 5 – 6 | 20.5 |  | 31.2 |  | 35.4 |  | 30.4 |  | 22.3 |  | 26.8 |  | 33.6 |  | 30.6 |  |
| 7 – 8 | 21.9 |  | 34.2 |  | 34.8 |  | 29.7 |  | 24.3 |  | 32.9 |  | 33.3 |  | 33.5 |  |
| 9 – 10 | 25.5 |  | 37.7 |  | 46.2 |  | 23.6 |  | 31.0 |  | 36.1 |  | 44.3 |  | 25.7 |  |
|  |  |  |  |  |  |  |  |  |  |  |  |  |  |  |  |  |
| NZiDep score |  |  |  |  |  |  |  |  |  |  |  |  |  |  |  |  |
| 1 | 18.8 | 0.0001 | 28.1 | 0.09 | 36.6 | 0.04 | 24.0 | 0.77 | 20.2 | <0.0001 | 26.3 | 0.004 | 32.6 | 0.004 | 23.1 | 0.24 |
| 2 | 18.8 |  | 35.2 |  | 40.3 |  | 23.5 |  | 22.9 |  | 28.1 |  | 39.5 |  | 24.7 |  |
| 3 | 27.0 |  | 37.3 |  | 42.4 |  | 27.5 |  | 28.7 |  | 36.7 |  | 40.9 |  | 37.0 |  |
| 4 | 29.4 |  | 34.3 |  | 52.7 |  | 20.7 |  | 30.9 |  | 34.9 |  | 49.0 |  | 26.7 |  |
| 5 | 27.6 |  | 42.2 |  | 44.7 |  | 9.1 |  | 31.2 |  | 39.8 |  | 43.4 |  | 30.0 |  |
|  |  |  |  |  |  |  |  |  |  |  |  |  |  |  |  |  |
| NZiDep score, binary | |  |  |  |  |  |  |  |  |  |  |  |  |  |  |  |
| 1 – 2 | 18.8 | <0.0001 | 30.9 | 0.05 | 38.3 | 0.01 | 23.9 | 0.78 | 20.9 | <0.0001 | 27.0 | 0.0001 | 35.4 | 0.002 | 23.5 | 0.05 |
| 3 – 5 | 27.9 |  | 37.4 |  | 47.2 |  | 22.5 |  | 30.0 |  | 36.7 |  | 44.8 |  | 31.9 |  |

**Supplementary Table S5.2:** Percentage of 5602 children enrolled in the *Growing Up in New Zealand* longitudinal cohort study hospitalised for an infectious disease within aggregated single-combined ethnic groups, by measure of socioeconomic status (NZDep2013 and NZiDep).

|  | Aggregated single-combined ethnic group | | | | | | | | | | | | | | | |
| --- | --- | --- | --- | --- | --- | --- | --- | --- | --- | --- | --- | --- | --- | --- | --- | --- |
|  | **Euro/Other only** | | **Māori only** | | **Pacific only** | | **Asian only** | | **Māori + non-Pacific** | | **Pacific + non-Māori** | | **Māori + Pacific** | | **Euro/Other + Asian** | |
|  | **%** | **P** | **%** | **P** | **%** | **P** | **%** | **P** | **%** | **P** | **%** | **P** | **%** | **P** | **%** | **P** |
| NZDep2013 |  |  |  |  |  |  |  |  |  |  |  |  |  |  |  |  |
| 1 – 2 | 18.9 | 0.02 | 25.0 | 0.17 | 33.3 | 0.74 | 17.9 | 0.001 | 18.6 | 0.01 | 25.9 | 0.70 | 66.7 | 0.005 | 13.5 | 0.70 |
| 3 – 4 | 19.0 |  | 70.0 |  | 46.7 |  | 15.8 |  | 20.9 |  | 30.2 |  | 17.9 |  | 20.8 |  |
| 5 – 6 | 20.5 |  | 30.0 |  | 35.7 |  | 33.6 |  | 23.7 |  | 27.3 |  | 39.0 |  | 23.5 |  |
| 7 – 8 | 19.0 |  | 32.6 |  | 41.7 |  | 36.1 |  | 34.4 |  | 26.9 |  | 29.9 |  | 23.4 |  |
| 9 – 10 | 28.2 |  | 44.2 |  | 46.9 |  | 24.3 |  | 27.2 |  | 35.7 |  | 44.3 |  | 23.3 |  |
|  |  |  |  |  |  |  |  |  |  |  |  |  |  |  |  |  |
| NZiDep score |  |  |  |  |  |  |  |  |  |  |  |  |  |  |  |  |
| 1 | 18.7 | 0.01 | 37.8 | 0.51 | 37.4 | 0.17 | 25.4 | 0.36 | 22.6 | 0.04 | 26.6 | 0.45 | 34.2 | 0.62 | 16.9 | 0.29 |
| 2 | 19.8 |  | 34.3 |  | 45.5 |  | 22.9 |  | 22.2 |  | 29.2 |  | 41.3 |  | 27.3 |  |
| 3 | 23.0 |  | 42.9 |  | 42.4 |  | 40.7 |  | 33.9 |  | 40.0 |  | 40.0 |  | 26.7 |  |
| 4 | 30.3 |  | 37.9 |  | 53.9 |  | 23.5 |  | 27.4 |  | 37.0 |  | 46.0 |  | 33.3 |  |
| 5 | 25.5 |  | 56.0 |  | 47.9 |  | 12.5 |  | 34.6 |  | 37.9 |  | 37.2 |  | 0.0 |  |
|  |  |  |  |  |  |  |  |  |  |  |  |  |  |  |  |  |
| NZiDep score, binary | |  |  |  |  |  |  |  |  |  |  |  |  |  |  |  |
| 1 – 2 | 19.0 | 0.001 | 36.3 | 0.25 | 41.2 | 0.09 | 24.8 | 0.35 | 22.5 | 0.004 | 27.6 | 0.06 | 37.0 | 0.38 | 19.4 | 0.42 |
| 3 – 5 | 25.9 |  | 45.3 |  | 49.0 |  | 30.8 |  | 31.5 |  | 38.5 |  | 41.6 |  | 25.8 |  |
